# Tramways and Suburban Traffic

**Published:** 1903-08-01

**Authors:** 


### Tramways and Suburban Traffic.

The London County Council has lately succeeded in affording an illustration of the great difficulty and complexity of some of the questions connected with London and suburban traffic. It has completed and opened three lines of electric tramways, which start from Blackfriars, Waterloo, and Westminster Bridges, unite at St. George's Circus and at Kennington, and thence diverge to Camberwell on the one side, and to Clapham, Balham, and Tooting on the other. The avowed object of these lines is to give working people easy access to suburban residences, and thus to remedy the evils inseparable from the costliness and the overcrowding of a great city. In many respects the experiment must be regarded as a success. The cars are large, airy, and frequent. They run at a speed which at present is still controlled by the presence of horse-cars upon the same metals, but which promises to become such as adequately to economise the time of the passengers, and to allow of the performance of a somewhat long journey at a rate approaching twelve miles an hour. The fares are very low, and between Tooting and the bridges there are no less than nine sections, each of which may be travelled over for one halfpenny; while for threepence the traveller is carried all the way. Working men and women are conveyed by the morning and evening cars at still lower rates; and there is an all-night service between the bridges and the Plough Inn, Clapham Common, the fare for which is only a penny. The natural crown of the undertaking would be furnished by the upspringing, at Balham and Tooting, of great towns of workmen's dwellings, towns fostered by the Council, and protected by its care from all the evils by which the physical welfare of dwellers in the slums of great cities has hitherto been imperilled or destroyed. The dreams of Fourier or of Zola were expected to be realised in the nearer suburbs of London; and the fragrant memories of the "progressive" members of the County Council were to be held in honour even by the remote descendants of all those who derived benefit from their works.

Robbie Burns, however, has taught us the fate which sometimes attends upon the "best-laid schemes of mice and men," and those of the Council have furnished no exception to his rule. Although not composed of electricians, but acting, we must presume, "under advice," the authorities in Spring Gardens determined to be the pioneers in this country, of a form of electrical traction known as "the conduit system," in which the cars receive their current from a cable situate in an underground channel between the rails. This channel is capacious, and of a contour which lends itself to sonorous vibration. The effect is that the approach of a car is heralded by a distant sound as of muffled drums, rising gradually to a roar like that of Niagara, and as gradually passing away. Each car, moreover, is furnished with a gong, actuated by the foot of the driver, and used to indicate its start after every stoppage, to give warning of its approach to horse-drawn vehicles in front of it, to afford an instrument for courteous salutation between drivers of cars that meet as they traverse the up and down lines respectively, and sometimes, to all appearance, as a mere outlet for lightsomeness of heart, like that of the ploughboy, who whistled as he went for want of thought. It is a well-established physiological fact that the human ear soon becomes accustomed to continuous sound, and that tourists sleep well within hearing of Niagara. Unfortunately, the roar of the cars is not continuous, and an intermittent Niagara, accentuated by gongs, cannot be described as a soporific. Beyond the limits of the Plough, where night cars cease their troubling, it might be thought that sleep would be undisturbed; but, in truth, it is murdered as effectually as ever it was by Macbeth. The night is utilised for the education of unskilled drivers. Mr. Grossmith used to describe in a lecture his endeavours to sleep at a Swindon hotel, in a room overlooking a yard in which young locomotives were taught to whistle; and his brief and sad experience is afforded every night to the residents on the main road between Clapham and Tooting. There is every prospect that, before long, the traveller from London to the latter place will pass through an intermediate belt of uninhabited country, and that the borders of the Balham and Clapham high-roads will return to prairie value, by a process the reverse of that growth of "unearned increment" which the Council has been so solicitous to tax. "Eligible premises" and "commodious residences," in any quantity, have been already placed upon the books of house-agents, but the business done in them has been but trifling in amount. It behoves the inhabitants of other districts, through which "conduit tramways" may be projected, not only to be warned and to be wise, but also to be wise in time.

